# Effect of Sol–Gel Silica Matrices on the Chemical Properties of Adsorbed/Entrapped Compounds

**DOI:** 10.3390/gels10070441

**Published:** 2024-07-02

**Authors:** Ariela Burg, Krishna K. Yadav, Dan Meyerstein, Haya Kornweitz, Dror Shamir, Yael Albo

**Affiliations:** 1 Chemical Engineering Department, Sami Shamoon College of Engineering, Beer-Sheva 84100, Israel; arielab@sce.ac.il (A.B.); krishphy25@gmail.com (K.K.Y.); 2 Chemical Sciences Department and The Radical Research Center, Ariel University, Ariel 40700, Israel; hayak@ariel.ac.il; 3 Chemistry Department, Ben-Gurion University, Beer-Sheva 8410501, Israel; 4 Nuclear Research Centre Negev, Beer-Sheva 9001, Israel; 5 Chemical Engineering Department and The Radical Research Center, Ariel University, Ariel 40700, Israel

**Keywords:** sol–gel, silica, adsorption, matrix inertness, hybrid matrices

## Abstract

The sol–gel process enables the preparation of silica-based matrices with tailored composition and properties that can be used in a variety of applications, including catalysis, controlled release, sensors, separation, etc. Commonly, it is assumed that silica matrices prepared via the sol–gel synthesis route are “inert” and, therefore, do not affect the properties of the substrate or the catalyst. This short review points out that porous silica affects the properties of adsorbed/entrapped species and, in some cases, takes an active part in the reactions. The charged matrix affects the diffusion of ions, thus affecting catalytic and adsorption processes. Furthermore, recent results point out that ≡Si-O. radicals are long-lived and participate in redox processes. Thus, clearly, porous silica is not an inert matrix as commonly considered.

## 1. Introduction

Sol–gel matrices are used for a variety of applications [1,2] of special importance for separations [3,4,5] and catalysis [6,7,8,9,10], including electrocatalysis [11,12]. The sol–gel process at the molecular level implies the ability to control the monomer → oligomer → sol (colloidal solution) → gel transitions. Thereafter, consider a porous silicon oxide material with the required chemical properties and surface morphological characteristics. The inner porosity of the resulting silica-based material enables accessibility, dispersion, and effective confinement of the entrapped molecular species. Three approaches are generally used for the preparation of sol–gel matrices:

1. Gelation of a solution of colloidal powders;
2. Hydrolysis and poly-condensation of alkoxide precursors followed by hypercritical drying of the gels;
3. Hydrolysis and poly-condensation of alkoxide or chloride precursors followed by aging and drying in ambient atmospheres.

Generally, chlorides or metal alkoxides are used as precursors for the preparation of the matrices through hydrolytic or non-hydrolytic processes [13]. The non-hydrolytic sol–gel process is based on the reaction between the chloride precursors with ethers/alcohols as oxygen donors as below (reaction (1)).

M-Cl + ROR/M-OR → M-OR/M-O-M + R-Cl (M = metal, e.g., Ti, Zr, Al, R = alkyl residues)(1)

The non-hydrolytic sol–gel synthetic route is particularly useful for the preparation of mixed oxides, as it enables excellent control over the homogeneity and the texture of the matrices obtained [14,15,16,17]. Compared to the matrices prepared via the hydrolytic process, these ones do not include hydroxyl groups. Instead, the residual surface groups are chlorides, which impart different characteristics to the oxide surface that affect the interactions with the immobilized species.

**Figure 1:**
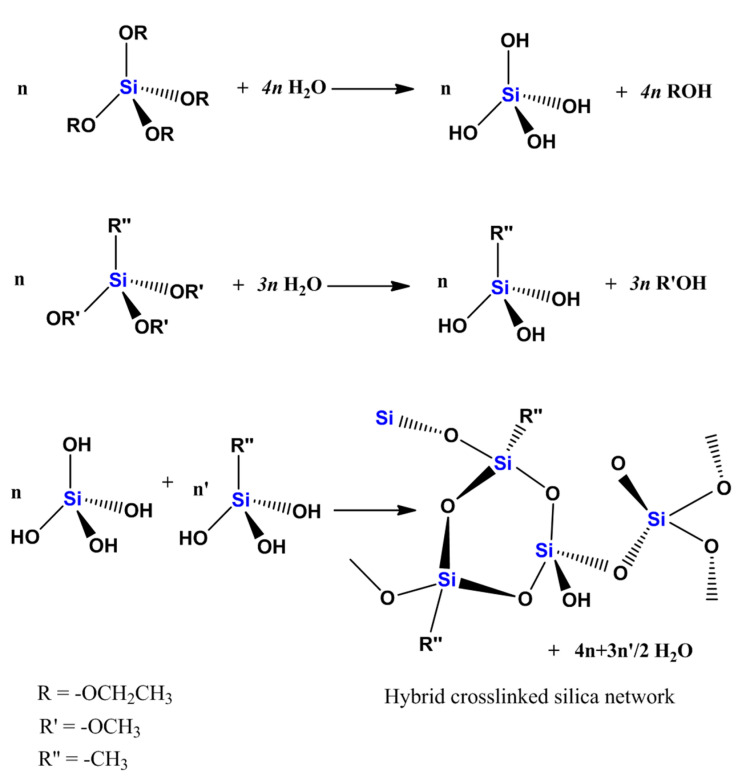
Synthetic pathway of silica sol–gel matrices.

The low cost (economics) and control over the end-product composition are important advantages of the sol–gel process. The silica-based matrices are usually prepared by using tetramethyl orthosilicate (TMOS) or tetraethyl orthosilicate (TEOS), which contains methoxide (-OCH_3_) and ethoxide (-OC_2_H_5_) groups [18,19,20,21] as a primary network-forming agent, allowing for a high degree of control over synthesis conditions, including pH, temperature, and the incorporation of additives. The synthesis of the sol–gel matrices involves hydrolysis and condensation steps in reactions that are outlined in Figure 1. The sol–gel chemistry of silica is typically driven by either acid or base catalysts, as the neutral reaction is very slow. The structure of the resulting gel is significantly different depending on the catalyst, and this is due to the relative rates of the hydrolysis and condensation reactions [22]. Organically modified silica (ORMOSIL) matrices can be prepared by using silane precursors of the type (RO)_3_Si-R′, where R′ contains the desired substituent [23]. Then, (RO)_3_Si-R′ is mixed with (OR)_4_Si, and the matrix is prepared through hydrolysis and condensation stages. The use of precursors of the type (RO)_3_Si-R′, where R′ has a functional substituent to which a desired compound can be covalently bound or itself has a role in the final matrix network [24,25,26], enables the control of the functionality and hydrophobicity of the matrix obtained [27,28,29]. These materials boast high specific surface areas (SSAs) exceeding facile formation and functionalization, tunable pore structures, and thermal stability [30]. While high-SSA silica has demonstrated functional effectiveness, continued research and development are essential to addressing evolving energy and environmental challenges.

Silica-based sol–gel materials synthesized with entrapped organic molecules, metal complexes, and metal nanoparticles (NPs) were extensively studied in the late 1980s. Early research involving the sol–gel process was conducted by D. Avnir and R. Reisfeld in 1984, who proposed to introduce a dopant solution (a dye molecule) at the preliminary stage of a silicon alkoxide gelation process and studied the activity of the silica resulting from mild drying of the intermediate alcogel [31]. They later demonstrated that any kind of organic species could be entrapped and well dispersed inside the pores of such silica materials, along with the complete retention of the chemical activity and considerable stability of the entrapped molecule [32]. The substrates and/or the catalyst are entrapped [33] in the matrix and/or adsorbed on its surface [34].

Commonly, silica matrices prepared via the sol–gel synthesis route are used as the matrix is “inert” and, therefore, is assumed not to affect the properties of the substrate or the catalyst [33]. This is clearly not accurate; adsorption means that there is an adsorption energy that clearly affects the properties of the adsorbate. Even entrapped species that in principle do not interact with the walls of the matrix are affected by the entrapment. First, the volume of the solvent in the pores is small, and thus their solvation/hydration is affected [35,36,37]. Furthermore, if the solvent is water, then the silanol groups on the surface have a pKa. For silanols, the point of zero charge (PZC) is ca. pH 4. Thus, the entrapped species is exposed to an electrical field if the pH differs from the PZC. Furthermore, the charges on the walls of the pores are expected to affect the diffusion of charged species towards the entrapped catalyst [38,39]. Two classic examples of these effects are: I. Avnir et al. have shown that an organo-metallic catalyst that is entrapped in a silica sol–gel matrix is active even if the solvent is water, though it is unstable in aqueous media [32]. II. Avnir et al. have shown that an enzyme entrapped in a silica sol–gel matrix is active at pHs and temperatures at which it denatures in homogeneous aqueous media [40].

This short review aims to highlight the effect of an “inert” porous matrix on the properties/reactivity of the adsorbate. The following sections discuss some specific effects of porous silica on the properties/stability of adsorbates and reaction mechanisms.

## 2. Cation Adsorption/Separation

Many human processes, such as industrial activity and the hazardous wastes it generates, cause environmental pollution. Among the most pervasive contaminants are toxic metals (e.g., Pu, U, Sr, Cs, Pb, Pb, Cd, Cr, and Hg), a particularly widespread group of pollutants with dire consequences for all animal life. The ubiquity of heavy metals in the environment can be traced to industrial development in parallel with the growing world population. A variety of physicochemical methods have been suggested to achieve the goal of reducing the environmental concentrations of toxic pollutants that are already imposing a crippling burden on human health. Among these methods are precipitation [41], filtration and membrane separation [42], reverse osmosis [43], and electrochemical [44] and biological processes [45,46]. Alternatively, adsorption can be used for the purification of wastewater from polluting metal cations. This method enables flexibility and can be easily implemented. Different adsorbing materials have been studied for this purpose, with the aim of finding cost-effective adsorbing materials with high capacity [47,48,49,50], selectivity, kinetic efficiency, and stability.

**Figure 2:**
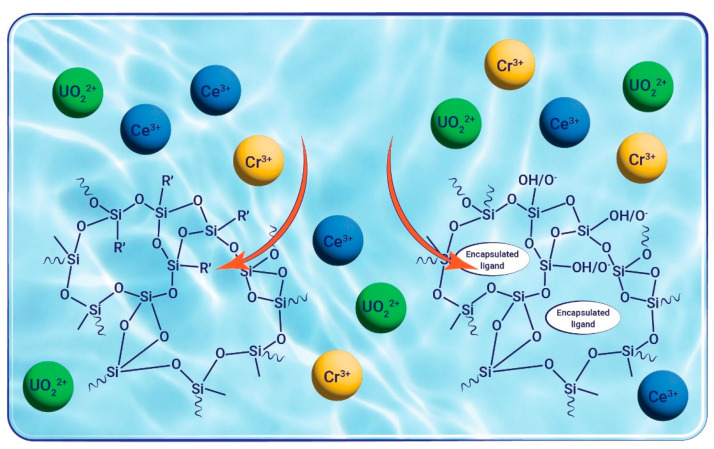
Sol–gel matrices function as adsorbing material for polluting metal cations by incorporating ligands into the matrix. Left side: organically modified matrices with R′ moiety that can coordinate to a metal cation e.g., R′ = C_6_H_5_,(CH_2_)_3_NH_2_; (CH_2_)_3_CN. Right side: silica matrix doped with ligands, e.g., DTPMP or NTPH.

Expanding the sol–gel methodology to prepare gel materials for metal ion extraction necessitates careful consideration of potential perturbations to complexation and, most importantly, the matrix pore structure, surface area, pH, and hydrophobic nature. Variability in pore size and distribution can impact the efficiency of metal absorption, as it may affect the accessibility of metal ions to active sites within the material. Additionally, the formation of large or irregular pores may lead to decreased surface area and reduced metal uptake capacity. Silica gels modified with complexing agents have also proven their effectiveness in extracting metals from aqueous samples [4,51,52,53,54,55]. These silica-based sol–gel materials offer distinct advantages, such as rapid exchange kinetics [4,56] and robust physical stability [57,58], distinguishing them from conventional functionalized resins. The identity of the target heavy metal for extraction dictates the precursors that are used in the matrix preparation. Silane precursors of the type (RO)_3_Si-R′, where R′ contains the desired substituent that can ligate to metal cations or can be used to bind ligands that are known to be selective to certain metal cations [59,60,61,62,63,64], can be used for the preparation of matrices with higher capacity and selectivity. Alternatively, ligands can be encapsulated in the matrix [65,66] by their addition at different stages of the sol–gel process (Figure 2). This allows for the precise control of both the kind of metal that will be bound and the strength of the binding that affects the regeneration efficiency. The specificity of the entrapment in terms of the target heavy metal cation can be achieved by rationally designing the matrix and taking into account hard and soft acids and bases considerations to enable the separation of metal cations in a common oxidation state.

As the silanol groups of the silica matrices are weak acids, they can act as ion exchange materials and separate cations from solutions. As the ≡Si-O^−^ is a hard, strong base, it prefers high-valent cations. Thus, though this was not reported, one can expect that the oxidation potentials of cations adsorbed to a sol–gel matrix are considerably lower than those in homogeneous aqueous media. The selectivity of gels for the adsorption of certain ions can be achieved by the addition of a complexing agent. For example, two experiments were performed for the selective binding of UO_2_^2+^. First, ethylenediaminetriacetate was covalently bound to a silica-gel matrix; the resulting matrix bounds both Ce^III^_aq_ and UO_2_^2+^_aq_ well, with a small preference for UO_2_^2+^_aq_ [67]. To improve selectivity, three kinds of matrices, which differ by the entrapped ligand (nitrilotris(methylene)]tris(phosphonic acid) [NTPH], diethylenetriaminepentakis (methylphosphonic acid) [DTPMP] and N1-(3-trimethoxy-silylpropyl) diethylenetriamine (N1)) were prepared [21]. In order to develop a method for uranyl separation from a solution containing a mixture of cations, phosphonate ligands, which are known to form stable complexes with uranyl [68,69,70,71,72,73], were chosen. The matrix that contained the DTPMP showed the highest capacities under most experimental conditions. However, the NTPH matrix was shown to have the best selectivity for cerium [21].

The results showed no selectivity for any of the cations that were studied with matrices prepared at basic pH, and no separation ability for those cations was obtained. However, a high capacity was found for the matrices that were prepared at pH 13 for all of the studied cations [21]. This result indicates that the sol–gel matrix could be applied as a method for durable and simple cation entrapment. The TMOS-Blank sol–gel matrix (without a ligand) was found to be selective for the adsorption of uranyl cations, and the addition of NTPH increased the matrix capacity and selectivity for cerium and accelerated the cation adsorption process. The density functional theory (DFT) calculations that were performed in order to explain the experimental results indicate that the uranyl (from the divalent cations) and chromium from the trivalent cations are bound to the matrix strongly, with and without NTPH, and as expected, the binding of trivalent cations is more exergonic than the divalent cations. Surprisingly, it was found by ^31^P-NMR and DFT calculations that the phosphonates, at least in part, were covalently bound to the matrix formed [21], probably via reaction (2):

≡Si-OH + R-PO_3_H^−^ → ≡Si-O-P(R)O_2_^−^ + H_2_O (2)

DFT calculations, at the level of PBEPBE/6-311+G(d,p) (SCRF = SMD; Empirical-Dispersion = GD3BJ) [74,75,76,77,78] using g16 software [79] were used to calculate the plausibility of the reaction of the NTPH ligand with the silica matrix (reaction (3)).

(OH)Si(OSi(OH)_3_)_3_ + N(CH_2_PO(OH)_2_)_3_ → NTPH_silica +H_2_O ΔG^0^ = −14.6 kcal/mol (3)

**Figure 3:**
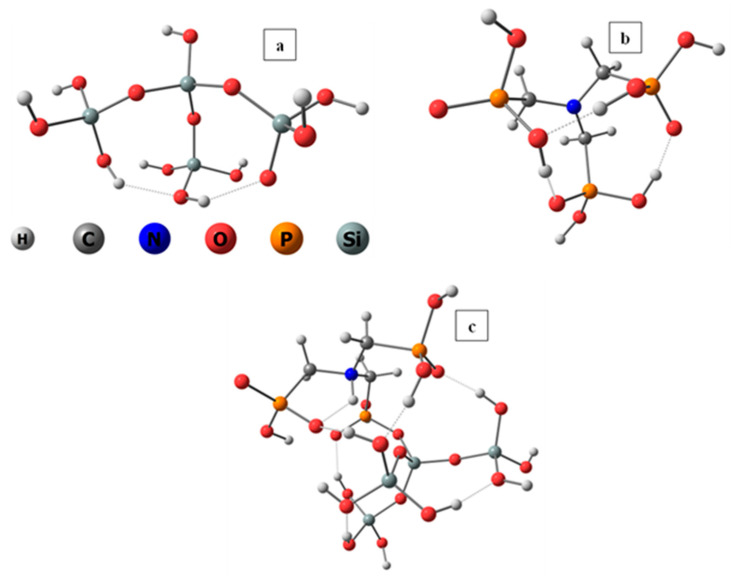
Ball and stick representations of (**a**) blank silica, (**b**) NTPH, (**c**) NTPH-entrapped silica.

This is an exergonic reaction, indicating that a variety of oxo-acids might be covalently bound to silica sol–gel matrices during their formation. In general, it is evidence that the sol–gel matrix is not inert. Figure 3 presents the structures used for the DFT calculations: the blank silica, the ligand, and the NTPH-entrapped matrix with the new bonds formed.

^29^Si and ^31^P solid-state NMR measurements were also used to study the structure and possible interaction between the silica network and the nucleic acids in DNA formed upon its encapsulation in a silica sol–gel matrix. While the ^29^Si NMR data did not indicate a possible bonding between siloxane chains and the DNA molecules, the ^31^P NMR spectrum showed that a complexation between the Si network and the DNA phosphate groups probably occurred through the formation of the P-O-C:(Si) link [80].

The formation of covalent bonds between the immobilized species and the silica matrices was also reported for the entrapment of dye molecules [81]. The absorption magnitude of the doped sol–gel films decreased with the increase in aging time, indicating changes in the interaction with the pore walls, and the conversion of indirect hydrogen bonds to direct hydrogen and covalent bonds by the elimination of water. Capeletti et al. have shown that the synthesis route affects the interaction formed between the encapsulated pH sensors, alizarin red, and the silica matrix. These interactions were studied by cyclic and differential pulse voltammetry. Matrices prepared via the base-catalyzed procedure showed differently shaped redox waves, thus indicating different interactions and/or the presence of other products formed during material preparation or in the cathodic/anodic scans [82]. Pereira et al. reported that C–OH bonds in polyvinyl alcohol were converted into C-O-Si bonds by esterification reactions occurring during the sol–gel process used to prepare the hybrid matrix [83,84], as observed by infrared spectroscopy.

### 2.1. Electron Exchange Columns

Electron exchange in which a redox agent bound to the solid packing matrix oxidizes or reduces one or more substrates passing through the column without releasing the entrapped moiety to the solution can facilitate the heterogenization and performance under the flow of numerous redox reactions that are highly relevant to a broad range of industrial and environmental remediation processes. Two types of electron exchange columns were prepared:

1. Oxidizing electron exchange columns by entrapping Ni^II^(cyclam)^2+^ in a sol–gel matrix [20] or by covalently binding Ni^II^(cyclam)^2+^ to a porous silica nanoparticle [85]. Transition metal complexes with high and low oxidation states are suitable redox active agents for heterogeneous electron exchange applications. The rational design of the ligand, i.e., altering the ligand structure to manipulate the redox potentials of the complexes, enables uncommon oxidation states of the metal to be stabilized. Both matrices were oxidized by S_2_O_8_^2−^ and then shown to oxidize reducing agents. The lifetime of the Ni^III^(cyclam)^3+^ oxidizing agent formed in these systems is considerably longer than in homogeneous media due to the inhibition of the reaction between two Ni^III^(cyclam)^3+^ complexes [85].
2. Reducing electron exchange columns by entrapping polyoxometalates (POMs). In recent years, POMs have attracted significant attention due to their alterable physical and chemical properties [86,87,88,89,90,91]. Moreover, they are known for their flexible redox behavior, which can be fine-tuned during the synthesis process by changing their composition [92,93,94]. The oxidized forms of POMs can accept electrons, whereas their reduced forms can function as the donors and the acceptors of several electrons while retaining their structures [92,95,96,97]. This property renders POMs ideal candidates for electron exchange applications [93,98,99,100]. Matrices prepared by the entrapment of PW_12_O_40_^3−^ and AlW_12_O_4_^5−^ in silica or organically modified silica by using the sol–gel procedure were used as reducing electron exchange columns [98,101]. The entrapped polyoxometalates were reduced by sodium borohydride, and the reduced product was shown to reduce halo-organic compounds [101] and bromate [102]. NMR studies proved that the polyoxometalates were bound covalently to the sol–gel matrix via a mechanism analogous to reaction (2). Also, the average number of electrons loaded on each silica-entrapped POM, *n*, was considerably smaller than that observed in experiments performed with POM dissolved in solution. Moreover, it depends strongly on the nature of the precursors. Higher values of *n* were obtained when matrices were more hydrophilic and prepared only from TEOS [17].

### 2.2. Electrocatalytic Processes by Entrapped/Adsorbed Species in Sol–Gel Matrices

Nickel [103] and ruthenium complexes [33] entrapped in sol–gel matrices were studied as electrocatalysts for water oxidation. A copper complex entrapped in sol–gel matrices was shown to be an efficient electrocatalyst for the heterogeneous de-chlorination of alkyl halides [104]. The results obtained in these studies point out that the precursors used to prepare the matrices dramatically affect the efficiency of the catalytic process. Thus, the use of trimethoxy-(phenyl)silane as one of the precursors considerably decreases the electrocatalytic current [33,104,105]. This is attributed to the hydrophobic properties of the pores induced by the phenyl groups in matrices prepared using a mixture of the silane precursors trimethoxymethylsilane (MTMOS) and trimethoxyphenylsilane and the steric hindrance they cause.

The results of the electrocatalytic water oxidation in the presence of a ruthenium complex and a co-catalyst, bicarbonate/carbonate, indicated that the current decreases in alkaline media [33], though it increases with pH when the same process is studied in homogeneous solutions [106]. This is attributed to the decrease in the diffusion coefficient of carbonate compared to bicarbonate in the negatively charged pores.

The study of the electrocatalytic de-halogenation in the presence of Cu(2,5,8,11-tetra-methyl-2,5,8,11-tetraazadodecane)^2+^ entrapped in a sol–gel matrix revealed three further effects of the matrix: I. The Cu(2,5,8,11-tetra-methyl-2,5,8,11-tetraazadodecane)^2+^ (Cu^II^L)-entrapped complex is stable even in acidic media. II. The redox potential of the entrapped complex is shifted somewhat cathodically, probably due to the charged pores. III. The mechanism of the de-halogenation in the heterogeneous system [105] differs from that in the homogeneous one [107,108]. In both media, the first step is:

RX + Cu^I^L^+^ → Cu^II^L^2+^ + R^.^ + X^−^ (X = Cl/Br) (4)

In homogeneous solutions, this is followed mainly by:

R^.^+ Cu^I^L^+^ → LCu^II^-R^+^; LCu^II^-R^+^ + H_2_O → Cu^II^L^2+^ + RH + OH^−^ (5)

Whereas in the heterogeneous system, the main follow up reactions are:

R^.^ + Cu^II^L^2+^ → LCu^III^-R^2+^; LCu^III^-R^2+^ + H_2_O → Cu^I^L^+^ + ROH + H^+^ (6)

The reason for the different mechanisms is that in the homogeneous system, excess Cu^I^L^+^ is present, whereas in the heterogeneous system, the radical is trapped in the pore in the presence of Cu^II^L^2+^ formed in reaction (4).

### 2.3. M^0^-NPs as Catalysts for Reduction Processes of Halo-Organic and Nitroaromatic Pollutants

The catalytic de-halogenation of halo-aliphatic compounds, e.g., haloacetic acids and chloroacetamides, by reduction with sodium borohydride in the presence of Ag^0^-NPs [109,110], Au^0^-NPs [110], and Fe^0^-NPs [111,112] entrapped in sol–gel matrices was studied. The results indicate that the de-halogenation rate and mechanism are affected by both the nature of the halo-organic substrate and the nature of the metal used to prepare the M°-NPs. The de-halogenation products of Br_3_CCO_2_H vary for M^0^ = Au^0^, Ag^0^, or Fe^0^. Nitrobenzene reduction with sodium borohydride was studied by comparing the catalytic performance of Fe^0^-NPs entrapped in organically modified silica matrix ZVI@ORMOSIL and Ni^0^ formed in situ by the reduction of Ni^2+^_aq_ adsorbed to a porous, organically modified silica matrix (Ni(II)@ORMOSIL) [113]. The question of whether the M^0^-NPs are adsorbed to the surfaces of the pores was not studied. The results clearly point out that the adsorbed Ni(II) is a better catalyst, and that the heterogeneous catalysis occurs via different reaction mechanisms compared to the reaction performed in the homogenous phase with Ni^2+^_aq_ as a catalyst. In the latter, no color change indicating the formation of Ni^0^ was observed, thus indicating that different catalytic species are formed, therefore demonstrating the effect of the matrix.

### 2.4. The Effect of Porous SiO_2_ on Catalytic Hydrogen Evolution Processes Induced by M^0^-NPs

The catalysis of water reduction in the presence of ^∙^C(CH_3_)_2_OH radicals obtained by γ irradiation of de-aerated aqueous solutions containing acetone and 2-propanol reactions (7)–(11) was studied for suspended M^0^-NPs and for silica-supported M^0^-NPs, M^0^-NPs@SiO_2_ (M = Ag; Au and Pt) [8,34,114].

n[(CH_3_)_2_COH.] + M^0^-NP → (M^0^-NP)-{C(OH)(CH_3_)_2_}_n_ (7)

(M^0^-NP)-{C(OH)(CH_3_)_2_}_n_ → (M^0^-NP)-{C(OH)(CH_3_)_2_}_n-m_^m−^+ m(CH_3_)_2_CO + mH^+^ (8)

(M^0^-NP)-{C(OH)(CH_3_)_2_}_n_^−^_m_^m−^ + kH^+^ ⇔ H_k_- (M^0^-NP)-{C(OH)(CH_3_)_2_}_n-m_^(m-k)−^ (9)

H_k_-(M^0^-NP)-{C(OH)(CH_3_)_2_}_n-m_^(m−k)−^ → H_k-2_-(M^0^-NP)-{C(OH)(CH_3_)_2_}_n-m_^(m-k)−^+H_2_ (10)

H_k_-(M^0^-NP)-{C(OH)(CH_3_)_2_}_n-m_^(m−k)−^ → H_k_-(M^0^-NP)-{C(OH)(CH_3_)_2_}_n-m-2_^(m−k)−^ + (CH_3_)_2_CHOH + (CH_3_)_2_CO (11)

The ratio [H_2_]/[(CH_3_)_2_CHOH] in the products increases with the negative charge on the nanoparticle, H_k_-(M^0^-NP)-{C(OH)(CH_3_)_2_}_n-m_^(m-k)−^. Silica support of the M^0^-NPs was shown to decrease this ratio, i.e., to catalyze reaction (9). This was interpreted as indicating a negative charge transfer from the silica to the M^0^-NPs [8,34,114].

In another study, it was shown that silica-supported silver nanoparticles, SiO_2_-Ag^0^-NPs, catalyze the hydrolysis of BH_4_^−^ [8]. A comparison of the isotopic composition of the hydrogen formed in the hydrolysis of BD_4_^−^ points out that the contribution of hydrides via the Heyrovsky mechanism, rather than that of hydrogen atoms through the Tafel mechanism, to the hydrogen evolution is considerably larger for the SiO_2_-Ag^0^-NPs catalysis process than for the Ag^0^-NPs catalysis process. This indicates the partial electron transfer from the SiO_2_ to the silver that increases the negative charge on the Ag^0^-NPs.

These results clearly point out that porous silica is not inert. The partial electron transfer from the silica to supported M^0^-NPs, which are not strong oxidizing agents, raises the question of whether one can oxidize porous silica surfaces.

### 2.5. Formation of ≡Si-O^.^ Radicals on Porous Silica Surfaces

Recent results point out that ≡Si-O. radicals [115] and probably other radicals, e.g., ≡Si-OO^.^; ≡Si-OOO^.^; ≡Si^+^-O_2_^.^^−^, are formed when H_2_O_2_ is adsorbed on silica surfaces [116,117]. The question whether the formation of these radicals is initiated by traces of iron present in silica is still debated [118]. In another recent study, long-lived ≡Si-O^.^ radicals were reported to oxidize sulfhydryls. OH^.^ and H_2_O^.^^+^ “are previously known to exist at water interfaces [119]”. In another study, it was proposed that OH^.^ radicals and H_2_O_2_ are formed “in aqueous microdroplets or at a water vapor–silicate interface [120]”.

Recently, a sol–gel matrix was used as an electron exchange matrix (EEM) for the oxidation of para-chloroaniline (PCA), a common pollutant in the pharmaceutical industry [3]. The DFT results pointed out the formation of ≡Si-O^.^ radicals by the reaction of the sol–gel silica with S_2_O_8_^2−^. The radicals formed react with the PCA to form radicals on the nitrogen atoms (reaction (12)). The DFT calculations ruled out the formation of hydroxyl radicals (reaction (13)).

Si_4_O_12_H_9_O^.^ + PCA → Si_4_O_12_H_9_(N^.^)C_6_H_4_Cl + H_2_O  ΔG° = −19.31 kcal/mol (12)

Si_4_O_12_H_9_O^.^ + PCA → Si_4_O_12_H_9_NHC_6_H_4_Cl + ^.^OH  ΔG° = 6.29 kcal/mol (13)

When Cu(II) was entrapped in the sol–gel matrix, its binding to the matrix was strong (reaction (13)), and the ΔG^0^ was affected by the pH of the PCA solution; as the pH increased, the reaction became more exergonic (reaction (14)) [3].

Si_4_O_13_H_10_ + Cu(II)(H_2_O)_6_^2+^ → Si_4_O_12_H_9_OCu(II)(H_2_O)_4_^+^ + H_3_O^+^ + H_2_O ΔG° (pH 0) = 5.74 kcal/mol, ΔG° (pH 2) = 3.01 kcal/mol, ΔG° (pH 7) = −3.81 kcal/mol, ΔG° (pH 13) = −12.01 kcal/mol(14)

The results, which are supported by DFT calculations, show that the silicon skeleton of the EEM has two important roles, both as a porous matrix that hosts the redox species and as an oxidant species involved in the advanced oxidation process.

## 3. Concluding Remarks and Future Perspectives

The sol–gel process enables the preparation of silica-based matrices with tailored composition and properties that can be used in a variety of applications, including catalysis, controlled release, sensors, and separation. The inclination towards the sol–gel process is primarily due to the high purity of the compounds, homogeneity, cost-effectiveness, and lower processing temperatures as compared to other traditional glass melting or ceramic powder methods. Clearly, recent results point out that porous silica not only affects the properties of compounds adsorbed to it but is also involved in the reactions of reactive oxygen species due to the formation of ≡Si-O. radicals and probably other radicals, i.e., it is involved in redox processes. Also, it is shown that in hybrid silica matrices prepared by the sol–gel process, in some cases, the interaction between the host matrix and the encapsulated species is stronger than van der Waals interactions, and covalent bonds are formed during the hydrolysis and condensation stages. The interactions of the adsorbed species with the sol–gel matrices are often not easy to detect due to their low content relative to the host matrix. Therefore, in any rational design of applicative matrices for catalysis, electron exchange columns, or adsorbing material for environmental applications, the interaction of the support with the adsorbed species and its effect on its activity should be considered.

## Data Availability

Not applicable.
